# Orthopædic Surgery—Remarks upon “Talipes”

**Published:** 1865-10

**Authors:** J. F. Miner


ART. III.—*Orthopædic Surgery—Remarks upon "Talipes."* By J. F. Miner, M. D.

Orthopædic Surgery is so little the common property of the profession, and so generally considered as belonging exclusively to those who cultivate this department as a specialty, that any attempt to make it general and popular will doubtless at present end in failure. The obstacles to be overcome in the treatment of deformities are numerous, and very formidable, arising both from the nature of the disease and from conditions not strictly or essentially connected with the disease itself. The leading minds in the profession who have bestowed attention upon this branch of surgical practice, have done so with the view that orthopædic institutions must be established in this country upon a basis similar to those in Europe, that "the principles of this inestimable science may be diffused far and wide," and have always seemed to direct their thoughts and efforts mainly to the erection of some great charity where this class of disease might receive exclusive attention.

That such an institution would afford advantages over private residences in the treatment of deformities, is sufficiently obvious, but that the principles of this inestimable science may not be diffused, and become the common property of the profession is by no means conceded. The surgeon in general practice cannot devote great attention to determining the obscure causes of muscular contraction, or why it should continue after causes producing it have been removed; the laws of reflex action may not be fully familiar to all the practitions of orthopædic surgery, if indeed the details of this action are even understood by any, but the general principles of this science so far as relief is concerned may be readily gained by every attentive practitioner of medicine; what is really known, even by those who have devoted to this subject great time and much patient labor, may be appropriated in its practical bearings, and in great degree made available in the general practice of medicine and surgery. These remarks are made as prefatory to what may be said upon the nature and treatment of deformities of the foot rather as encouragement to general practitioners to attempt restoration, and not to allow the generally received opinions that private treatment is inefficient and unsuccessful, to dissuade from well directed effort in the treatment of congenital or acquired deformities. An institution for the exclusive treatment of such disease might be a great charity, but a much greater and more wide-spread charity would be instituted, by practical knowledge of the principles of treatment, being made the common possession of the masses of the profession. That which has been neglected by the profession, has been seized by a herd of pretenders and quacks who claim as their divine gift, what medical art has failed to achieve. The sufferers, who gather around them by hundreds, are fleeced, duped, and deceived, and ignorantly suppose that their failure to obtain relief is due to impossibility of cure, rather than to the dishonesty and incapacity of the arrogant pretender they have consulted. There is no corner of the domain of medicine unentered by these pretentious villains, who are inexhaustible in their promises and efforts to delude the ignorant and credulous. Physicians should not avoid and neglect patients having deformity though they have passed the usual routine of quackish divination, and apply for relief after "laying on of hands, natural bone setting, angle-worm oil, magnetic baths, galvanic salve," and a host of other absurdities have failed to relieve a condition of disease, which is safely and certainly curable when given up to judicious and enlightened effort.

"*Talipes*" has been introduced as a generic term to all the distortions of the foot, with or without displacement of the articular surfaces of the tarsal bones; different mal-positions of the foot have received descriptive appellations. The foot is frequently the seat of mal-position, which is often congenital; the larger number however are said to be acquired. Some forms are almost always congenital, as club-foot for instance, while others rarely.

The causes of the various mal-positions of the foot cannot be briefly stated, if they could be positively determined at all; the causes of congenital deformity are unknown, though it may be said that many of the most stubborn facts favor the old and popular notion that impressions made upon the mind of the mother, may affect the fœtus in utero, which view has been recently discarded, and even ridiculed by "theoretical physiology." Acquired deformity may arise from various causes, but is produced generally by permanent contraction of muscles, which are of course obedient to the nervous system, reflecting changes which may have taken place in this system, or which have been communicated to it by reflex influence. Disease of the joints is a frequent cause of deformity, both from displacements and from contraction of muscles. Contraction of muscles is the almost uniform condition in cases of mal-position, the bones acting in obedience to the forces which are applied to them. Upon this fact rests many of the therapeutical and mechanical indications which are present in cases of deformity—the muscles are drawing or have drawn the bones from their natural positions, and to this fact whatever may have been the exciting or remote causes we are to direct attention.

In some cases the muscles are found to not only have displaced the bones, but to have become fixed themselves, and incapable of contraction or extension; this fact is of great importance in its relations to treatment. Upon what this fixed condition of the muscular fibre depends is not so obvious, but probably upon perverted nervous influence or changes in the muscular fibre itself, or both these causes combined; however this may be the fact remains, and the indications of treatment are based upon it. In recent cases this is not present, and extension may be resorted to with some confidence of success, while those of long standing should not be subjected to this torture, since it will certainly end in failure, and may even aggravate and complicate the disease.

Are the deformities of the foot capable of restoration, and if so, by what means? are questions which practical men ask, and desire answers in as direct manner as is consistent with a correct understanding of the reply. If a child is born with feet distorted it can be restored in great degree; it *can* be perfectly or almost perfectly restored, but with the usual attendance which parents bestow themselves or hire others to do for them, it rarely, if ever *is*, perfectly restored. The grounds of this failure are sufficiently obvious, and rest with the patient and friends; for the best surgeon without co-operation can rarely accomplish satisfactory results, unless assisted by the most energetic and hearty approval on the part of attendants and friends. In many cases partially turned feet can be replaced when the child is young—the younger the better—and made to grow into natural form; this can be done without division of tendons if the effort is made with determination, and the retentive appliances efficient. Great deformity will require division of all of the tendons of contracted muscles, and failure is often from neglect of this; the tendo achillis being accused of all trouble, when in reality other muscles are equally in fault.

Restoration of deformed feet then, require, if recent, to be replaced forcibly in right position and retained; when distortion is slight, this may often be effected without division of tendons. If the deformity is great, the contracted tendons should all be divided, and foot placed immediately in proper position; delaying to dress, for a few days after division of the tendons, is believed to be wholly unnecessary. Inflammation to any troublesome or dangerous extent is exceedingly rare, and never greater by immediately placing the foot where it is to be retained. Fears are entertained by patients and sometimes by physicians, that tendons divided will not unite, and that thus will be produced permanent weakness. Upon this point of union there need be no fears, they will invariably unite and become strong; the weakness which is apt to be present in such cases, is due wholly to other and often to obvious causes—atrophy of muscles, perverted nervous influence, disuse and other influences in no way connected with the division of tendons. Division of greatly contracted tendons is indispensable to perfect success in the treatment of club-foot, and without this, the great majority of these deformities will remain unimproved, whatever other treatment may be adopted.

What retentive apparatus is best suited to our purposes in the treatment of deformities of the foot? Upon this point there will be found diversity of opinion; one, using some instrument, and another applying some form of retentive dressing—club-foot boots, of various patterns, paste-board, sole-leather, starch bandage, India-rubber, plaster paris, adhesive plaster, etc., etc., constituting the staples from which surgeons have selected to suit themselves. Adhesive plaster is well suited to answer the indications, and may be so applied as to fulfill all the objects desired in retention of feet which have been badly turned. The objections to its use are, mainly, that it requires too much time and attention of the surgeon; but this objection holds almost equally with all efficient means. Whoever trusts in the first of the treatment, to shoes made for the purpose, or other instrument to be applied by others than himself, will almost certainly fail of accomplishing the results he expects—will be disappointed in his just expectations. Sole leather may be easily converted into most admirable splints, and perhaps is unsurpassed for many purposes as a retentive material; the manner of preparing it, is doubtless familiar to all. Plaster paris has been highly recommended; as a material for stiffening bandages it may have some advantages over starch, and be made useful in the absence of more pleasant dressings.

Instruments fitting to properly made boots are convenient in the later months of treatment, but at first are more often injurious than otherwise. In conclusion it should be remarked, that success depends upon conditions and influences which the surgeon cannot always control; determination and perseverance are, however, essential, for the best of results can never be obtained without a rigid and protracted retention of the foot in natural form; it should be retained until it has time to grow into such position.

